# Biogeographical Variation and Population Genetic Structure of *Sporisorium scitamineum* in Mainland China: Insights from ISSR and SP-SRAP Markers

**DOI:** 10.1155/2014/296020

**Published:** 2014-03-17

**Authors:** Liping Xu, Yunhai Lu, Qian You, Xiaolan Liu, Michael Paul Grisham, Yongbao Pan, Youxiong Que

**Affiliations:** ^1^Key Laboratory of Sugarcane Biology and Genetic Breeding, Ministry of Agriculture/Fujian Agriculture and Forestry University, Fuzhou 350002, China; ^2^USDA-ARS, Sugarcane Research Unit, Houma, LA 70360, USA

## Abstract

A total of 100 *Sporisorium scitamineum* isolates were investigated by inter simple sequence repeat (ISSR) and single primer-sequence related amplified polymorphism (SP-SRAP) markers. These isolates were clearly assorted into three distinct clusters regardless of method used: either cluster analysis or by principal component analysis (PCA) of the ISSR, SP-SRAP, or ISSR + SP-SRAP data set. The total gene diversity (*H*_*t*_) and gene diversity between subpopulations (*H*_*s*_) were estimated to be 0.34 to 0.38 and 0.22 to 0.29, respectively, by analyzing separately the ISSR and SP-SRAP data sets, and to be 0.26–0.36 by analyzing ISSR + SP-SRAP data set. The gene diversity attributable to differentiation among populations (*G*_*st*_) was estimated to be 0.35 and 0.22, and the gene flow (*Nm*) was 0.94 and 1.78, respectively, when analyzing separately ISSR and SP-SRAP data set, and was 0.27 and 1.33, respectively, when analyzing ISSR + SP-SRAP data set. Our study showed that there is considerable genetic variation in the analyzed 100 isolates, and the environmental heterogeneity has played an important role for this observed high degree of variation. The genetic differentiation of sugarcane smut fungus depends to a large extent on the heterogeneity of their habitats and is the result of long-term adaptations of pathogens to their ecological environments.

## 1. Introduction

China is the second largest consumer of sugar and the third largest producer internationally [1]. Sugarcane (*Saccharum *complex) is the most important sugar-yielding crop in China and accounts for about 92% of sugar output [1]. It has become a more important economic crop due to an increase in sugar demand. Furthermore, sugarcane offers the potential to be an ideal bioenergy crop because it can produce readily fermentable sugars, high fiber content, and very high yields of green biomass [1]. Sugarcane smut caused by* Sporisorium scitamineum* is one of the most intractable and devastating diseases of sugarcane in the world [2–7]. It causes not only considerable yield loss, but also leads to variety elimination due to susceptibility to the disease [8]. At present, the most economical and efficient way to control the smut disease is to plant resistant cultivars. Prior to the 1940s, sugarcane smut was limited to Asia, a few countries of South and East Africa (Natal, Mozambique, and Zimbabwe), and the Mascarenes (Madagascar, Mauritius, and Reunion) [9]. Since then, smut has spread to almost all sugarcane producing countries. In China, smut has been the most prevalent disease in the last ten years due to the susceptibility of the sugarcane cultivar ROC22 which occupies more than 50% of the total sugarcane planting area. The average of smut infection rate in ROC22 is over 8% in plant cane and 15% in ratoon cane, respectively (personal communication).

The smut-resistant mechanism of sugarcane has been widely investigated at the morphological, physiological, and mainly molecular level [1–3]. Several kinds of molecular techniques, including cDNA-amplified fragment length polymorphism (cDNA-AFLP) [10–13], differential-display reverse transcription-PCR (DDRT-PCR) [14], cDNA microarray [15], Solexa sequencing [16], and two-dimensional gel electrophoresis (2-DE) coupled with matrix-assisted laser desorption ionization-time of flight mass spectrometry (MALDI-TOF-TOF/MS) [17], have been occupied for the researches on the molecular interaction between sugarcane and* S. scitamineum*. The investigation of the genetic diversity of* S. scitamineum* is essential for a better understanding of the structure of pathogen populations and the interactions among host and pathogen and the environment and as a basis for developing smut-resistant sugacane varieties. Molecular detection of the smut pathogen in sugarcane was firstly realized by using polymerase chain reaction (PCR) and then TaqMan Real-time PCR to amplify the bE mating-type gene of* S. scitamineum* [5, 18, 19]. The intraspecies diversity within* S. scitamineum* isolates has been studied by the methods of random amplification of polymorphic DNA (RAPD) [6, 20], amplified fragment length polymorphism (AFLP) [4], and internal transcribed spacer (ITS) sequence analysis [21, 22]. Raboin et al. analyzed a collection of* S. scitamineum* populations from 15 sugarcane producing countries for polymorphisms at 17 microsatellite loci [9]. The authors found that the genetic diversity was extremely low among the American and African populations but was high among the Asian populations. They also found that the American and African* S. scitamineum* populations all belonged to a single lineage which was also found among some Asian populations. In China, Que et al. studied the molecular evolution of* S. scitamineum* by analyzing a set of 23 isolates collected from six primary sugarcane producing areas in Mainland China with RAPD and sequence-related amplified polymorphism (SRAP) markers [7]. The results of RAPD, SRAP, and combined RAPD-SRAP showed all that the molecular evolution of* S. scitamineum* was associated with its geographical origin. No evidence of coevolution between the pathogen and sugarcane was found. These analyses also did not provide information about the race differentiation of* S. scitamineum*. Furthermore, the numbers of host genotypes, smut isolates, and geographical origin of districts in China for testing were limited to 21, 23, and 6, respectively, in the study of Que et al. [7], and the smut isolates studied by Raboin et al. [9] and Singh et al. [6] did not include the isolates from Mainland China. Thus, to better understand the smut population structure and the influence of environmental and genotype heterogeneity on the population structure, analysis of more isolates representing more regions, more cultivar/genotypes, and a representative sugarcane variety in different regions is needed.

Population genetic analysis of* S. scitamineum* could help to identify potential sources of resistance, which are expected in areas where genetic diversity of both pathogen and host is maximal [9]. The genetic diversity of* S. scitamineum* populations was shown to be extremely low among 142 single teliospore isolates collected from 15 countries based on the analysis of AFLP and RAPD markers [9]. ISSR markers are more reliable than RAPD because of the longer primers and the higher annealing temperature of ISSR than RAPD [23]. Therefore, the ISSR markers have been widely used in marker-assisted selection, genetic diversity analysis, or DNA fingerprinting [23–26]. Sequence-related amplification polymorphism (SRAP) technology was firstly developed from* Brassica *crops by Li and Quiros [27]. Currently, all SRAP markers are based on the use of a combination of upstream and downstream primer pairs [27]. In our previous research, the feasibility of single primer sequence related SRAP (SP-SRAP) markers have been proved for genetic diversity analysis (private bulletin).

In this study, we use ISSR and SP-SRAP techniques to study a more representative* S. scitamineum* isolate collection from Mainland China with the aim of understanding, at the molecular level, the differentiation, variation, and population structure of* S. scitamineum* in Mainland China and the effectiveness of the environmental and genotype heterogeneity on the influence of the pathogen population dynamics to provide the basis for* S. scitamineum* resistance breeding and the geographical distribution of varieties.

## 2. Materials and Methods

### 2.1. *S. scitamineum* Isolate Sampling and Isolation

**Figure 1:**
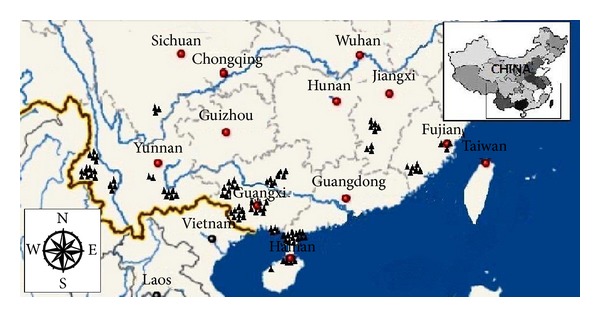
Geographic locations of the 100* S. scitamineum* isolates isolated from 7 Provinces of China. Notes: The seven provinces where the* S. scitamineum* isolates were collected are marked in the red dots.

One hundred pathogen strains were isolated in 2010 from 38 different sugarcane genotypes in 7 provinces (20 sugarcane planting districts) of China, including Guangxi (which accounts for 62% sugarcane planting area) (36 strains), Yunnan (26 strains) (accounts for about 20% sugarcane planting area), Guangdong (13 strains), Fujian (9 strains), Jiangxi (8 strains), Hainan (5 strains), and Sichuan (3 strains). More detailed information about these strains are given in Table 1 and Figure 1.

Single teliospores were isolated from whips of infected sugarcane with sterile tips or inoculation needle under the sterile conditions and separated by serial 10-fold dilution until only 1–5 single-teliospore(s) were observed in a single drop (10 *μ*L) under the microscope (40 × 10). 10–30 *μ*L of dilution was then evenly plated onto potato dextrose agar (PDA) medium, containing 75 mgl^−1^ streptomycin sulphate according to Yang et al. [28], then incubated for 1–3 days at 28°C. Germinating single teliospores were transferred to new plates containing PDA medium.

### 2.2. DNA Extraction and* S. scitamineum* Isolate Identification

Genomic DNA from hyphal colonies developed from single teliospores were extracted by the modified SDS method described by Que et al. [20]then stored at −20°C. The quality and quantity of extracted DNA were controlled on a 0.6% agarose gel. Electrophoresis was performed in 1 × TAE running buffer, pH 8.0, at 100 V. The agarose gel was checked with a NanoVue Spectrophotometer (GE Healthcare). DNA was quantified by fluorometry and adjusted to 20 ng/*μ*L in distilled water. In order to understand the relationship between single teliospores from the same smut whip, we randomly selected five single teliospores from one smut whip collected from the sugarcane cultivar ROC22 and cloned and sequenced the rDNA internal transcribed spacer (rDNA-ITS) regions (ITS1, ITS2, and 5.8S rDNA) as described by Singh et al. [6]. To ensure that all the DNA samples were from* S. scitamineum*, we amplified these DNA samples by* S. scitamineum* specific primers *b*E4 and *b*E8 [18], cloned the amplicons by T-vector, sequenced the cloned fragments, and compared them by BLASTn to the NCBI databases (http://www.ncbi.nlm.nih.gov/).

### 2.3. PCR Reactions, ISSR, and SP-SRAP Analysis

To ensure the identity of our* S. scitamineum* samples, we first performed PCR amplifications using the universal primers ITS1 and ITS4 for detection of fungi [29] and the specific primers *b*E4 and *b*E8 for detection of* S. scitamineum* [18].

For ISSR analysis, the primers used (P5, P6, P10, P21, P25, P26, and P27) were commercially synthesis and* Ex Taq* polymerase purchased from the TaKaRa Biotechnology Co., Ltd. (Dalian, China). Amplification reactions were performed in a total volume of 20 *μ*L containing 20 ng of DNA, 5 pmol of each primer, 4.5 nmol of each dNTP, 2.2 *μ*L of 10 ×* Ex Taq* Buffer, and 0.5 U* Ex Taq *polymerase. The final volume was adjusted to 20 *μ*L with sterile distilled water [8, 24]. The amplification cycles consisted of an initial denaturation at 95°C for 4 min, followed by 35 cycles of 30 sec at 94°C, 30 sec at 48–56°C, and 30 sec at 72°C, and a final extension phase of 10 min at 72°C. PCR products were separated by electrophoresis in 3.0% agarose gel with 1 × TAE buffer at 60 V for 3 h. The gels were stained with ethidium bromide and photographed under ultraviolet light with NanoVue Spectrophotometer (GE Healthcare).

For SP-SRAP analysis, the amplification reactions were performed in a volume of 25 *μ*L containing 2.4 *μ*L of 10 × Ex Tap Buffer, 5 nmol of each dNTP, 20 pmol of primer, 1.0 U* Ex Taq* polymerase, and 40 ng of DNA template. The amplification cycles consisted of an initial denaturation at 95°C for 5 min, followed by 5 cycles of 1 min at 94°C, 1 min at 35°C, and 1 min at 72°C, 35 cycles of 1 min at 94°C, 1 min at 35°C, and 1 min at 72°C, and a final extension phase of 10 min at 72°C. PCR products were separated by electrophoresis in 3.0% agarose gel with 1 × TAE buffer at 60 V for 3 h. The gels were stained with ethidium bromide and then photographed under ultraviolet light as above.

### 2.4. Data Analysis

The softwares of NTSYS.PC (Numerical Taxonomy System, Applied Biostatistics, Inc.) [30] and POPGENE1.31 were used for analysis of ISSR, SP-SRAP, and ISSR + SP-SRAP data. A binary matrix was firstly generated by scoring the presence or absence of each individual band in all lanes. A similarity matrix was secondly generated by using Jaccard coefficient. The Jaccard coefficient takes into account the bands present in at least one of the two compared individuals. The absence of bands is not interpreted as a similar character between isolates. The similarity matrix was used for unweighted pair-group method with arithmetic average (UPGMA) cluster analysis and principal component analysis (PCA) [30]. Observed number of alleles (*N*_*a*_), effective number of alleles (*N*_*e*_), Nei's measure of gene diversity (*h*), and Shannon's Information index (*I*) were used to evaluate the genetic diversity within each population by POPGENE1.31 [31, 32]. Mean values of Nei's gene diversity of total populations (*H*_*t*_), Shannon's diversity index between populations (*H*_*s*_), proportion of gene diversity attributable to differentiation among populations (*G*_*st*_), and estimates of gene flow parameter among populations *Nm* = 0.5(1 − *G*_*st*_)/*G*_*st*_  were obtained across loci [31, 32].

### 2.5. Sequence Analysis of ITS Regions

Based on results of ISSR and SP-SRAP analyses, somepairwise comparisons among isolates with low similarity values were selected for further investigation of ITS sequences (ITS1, ITS2, and 5.8S rDNA). The sequences were analysed by using the software DNAMAN (http://www.lynnon.com/) and the consensus sequences were compared by BLASTn to the NCBI databases (http://www.ncbi.nlm.nih.gov).

## 3. Results

### 3.1. Confirmation of* S. scitamineum* Samples

The DNA extracts of 100* S. scitamineum* isolates were firstly analyzed by NanoVue minim spectrophotometer. The ratios of OD_260_/OD_230_ ranged from 1.90 to 2.20 and the ratios of OD_260_/OD_280_ ranged from 1.60 to 2.10, indicating that all these DNA samples meet the requirement of the following experiments. To ensure the identity of our* S. scitamineum* samples, we performed PCR amplifications on these DNA samples using the universal primers ITS1 and ITS4 for specific detection of fungi [29] and the specific primer pair *b*E4 and *b*E8 for specific detection of* S. scitamineum* [18]. PCR amplification of the rDNA-ITS sequence using the specific primer pair ITS1 and ITS4 yielded a single 755 bp DNA fragment of expected size for each of the 100 DNA samples, extracted from the cultures of single-teliospores of the 100* S. scitamineum* isolates. Sequencing of five randomly selected amplified products showed a sequence similarity of >99.93% among them. PCR analysis of the 100 samples by* S. scitamineum* specific primer pair *b*E4 and *b*E8 revealed a fragment with expected size of 420 bp, which is present on all the tested samples. These fragments were cloned in TA-vector and sequenced. Sequence analysis showed that this fragment was a sequence specific to* S. scitamineum*.

### 3.2. Genetic Diversity of* S. scitamineum* Isolates by ISSR Analysis

Among the 18 ISSR primers tested on a set of five DNA samples, seven, namely P5, P6, P10, P21, P25, P26, and P27, were selected to amplify the DNA extracts of the 100* S. scitamineum* isolates. A total of 121 bands were obtained, of which, 105 were polymorphic among the 100 samples. The ISSR PCR amplification products and their levels of polymorphism are presented in Table 2. Each of these seven primers generated nine to 19 bands per isolate, which range in size from 250 bp to 5,000 bp. On an average, 17.3 bands were amplified by each primer, and the percentage of polymorphic bands was as high as 86.8% as estimated from the 100 isolates.

Based on the ISSR PCR amplification data, the genetic similarity indexes between the 100* S. scitamineum* isolates were calculated using Jaccard coefficient [30], and their values ranged from 0.46 to 0.90, with an average of 0.66. The maximum similarity coefficient of 0.9008 was obtained between the isolates from the hosts ROC16 (number 76) and GT98-296 (number 77), both of which were isolated in Fujian province. The minimum pairwise similarity coefficient of 0.46 was obtained between the isolates from the hosts GT11 (number 45) and CZ6 (number 98), of which the former was isolated in Yunnan province, and the latter was isolated in Sichuan province.

**Figure 2:**
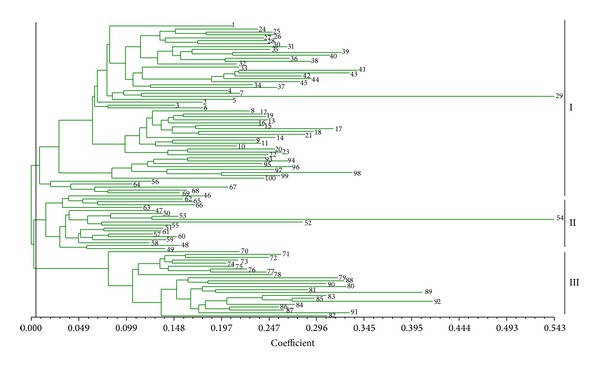
Dendrogram generated for the 100* S. scitamineum* isolates (numbers 1–100) based on ISSR data.

Based on the ISSR data set, a dendrogram was generated for the 100* S. scitamineum* isolates by unweighted pair-group method with arithmetic average (UPGMA) cluster analysis and is presented in Figure 2. They were grouped into three clusters at the level of 0.006, I, II, and III, respectively. The cluster analysis showed that most of these strains were relatively concentrated. Of the 100 isolates, 58 isolates (58%) were grouped in cluster I, including all the isolates collected in Guangxi, Hainan, and Sichuan, together with four isolates collected in Jiangxi and 10 isolates collected in Yunnan. Nineteen isolates (19%) were grouped into the cluster II, including 16 from Yunnan and 3 from Jiangxi. The remaining 23 isolates fell into the cluster III, including the 13 isolates from Guangdong and eight isolates from Fujian, plus one isolate from Jiangxi. These results suggest that the smut pathogen isolates collected from same region (province) tended to cluster in the same group.

**Figure 3:**
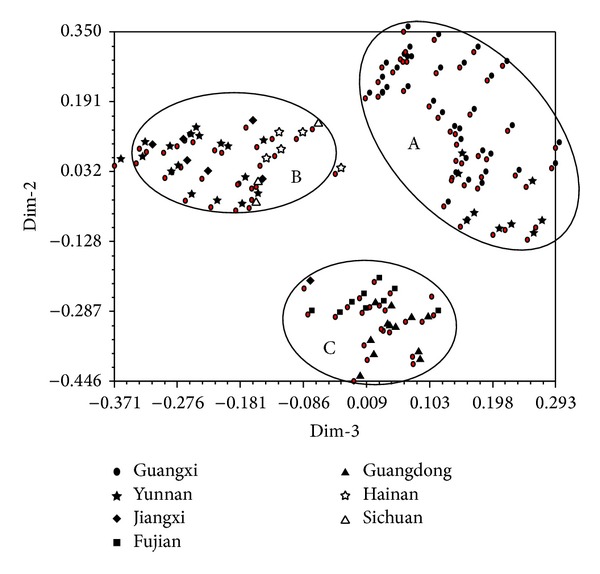
Principal components analysis using ISSR data for the 100* S. scitamineum* isolates (numbers 1–100).

A principal component analysis (PCA) based on the ISSR data set was performed. The first three components explained 97% of the total variance and divided clearly the 100 isolates into three groups, A, B, and C, as shown in Figure 3 by the dimension 2-3 plot. Group A includes 45 isolates, of which 19 are from Yunnan and 36 are from Guangxi. Group B includes 32 isolates, of which seven are from Yunnan, seven are from Jiangxi, five are from Hainan, and three are from Sichuan. Group C includes the remaining 23 isolates, of which 13 are from Guangdong, nine are from Fujian, and one is from Jiangxi. Compared to the results of UPGMA analysis, the 45 isolates assigned to group A by PCA belong to the cluster I using UPGMA, the 19 isolates assigned to group B by PCA are the same as those in the cluster II using UPGMA and the 23 isolates assigned to group C by PCA are the same as those in the cluster III using UPGMA. From the above analyses, the smut pathogen strains from a same region (province) tend to group into the same group. For example, the 36 isolates from Guangxi are clustered into a single group A, the five isolates from Hainan and the three isolates from Sichuan cluster in group B, and the 13 isolates from Guangdong and the nine isolates from Fujian clustering group C. Of the remaining 34 isolates, 19 of the 26 from Yunnan cluster in group A and seven into group B and seven of the eight isolates from Jiangxi cluster in group B.

### 3.3. Genetic Diversity of* S. scitamineum* Isolates by SP-SRAP Analysis

Among the 17 SP-SRAP primers tested on a set of five DNA samples, seven produced clear amplification patterns with bands ranging in size from 500 bp to 5,000 bps. The seven primers were used to amplify the DNA of the 100* S. scitamineum* isolates and yielded a total of 153 bands, of which 152 (99.3%) were identified as polymorphic. The number of polymorphic bands generated by each primer ranged from 9 to 27 with an average of 21.7. Except the primer P2, which produced one monomorphic band (of 10 bands) among the 100 isolates, the other 6 primers produced 100% polymorphic bands (Table 3).

The genetic similarity indexes generated from SP-SRAP data for the 100* S. scitamineum* isolates ranged from 0.44 to 0.79 with an average of 0.62. The maximum genetic similarity coefficient value was 0.7943 which was obtained from the following three compared isolate pairs: ROC22 (number. 7) and YT94-128 (number 19), ROC22 (number 18) and YT94-128 (number 19), and YT94-128 (number 19) and ROC22 (number 20). All 4 isolates were collected from Guangxi.

**Figure 4:**
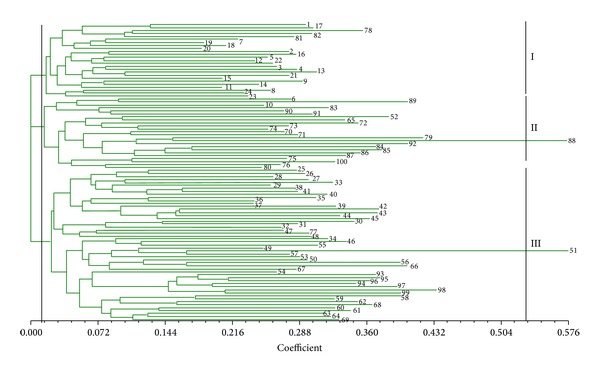
Dendrogram of the 100* S. scitamineum* isolates (numbers 1–100) generated by UPGMA based on SP-SRAP data.

UPGMA clustering analysis based on the SP-SRAP data set also divided the 100* S. scitamineum* isolates into three clusters, I, II, and III, at the distance index of 0.01 (Figure 4). The first cluster (I) consisted of 25 isolates, of which 22 were from Guangxi, two from Guangdong, and one from Fujian. The second cluster (II) contained 24 isolates, of which 11 were from Guangdong, seven from Fujian, two from Guangxi, two from Jiangxi, one from Yunnan, and one from Sichuan. The third cluster (III) was made up of 51 isolates, of which 25 were from Yunnan, 12 from Guangxi, six from Jiangxi, five from Hainan, two from Sichuan, and one from Fujian. It should be noted that the isolates from Hainan were grouped in the same cluster III, while the isolates from Guangxi and Fujian were distributed into all three clusters (I, II, and III), the isolates from Guangdong fell into clusters I and II, the isolates from Sichuan fell into clusters I and III, and the isolates from Jiangxi and Yunnan fell into clusters II and III, respectively.

**Figure 5:**
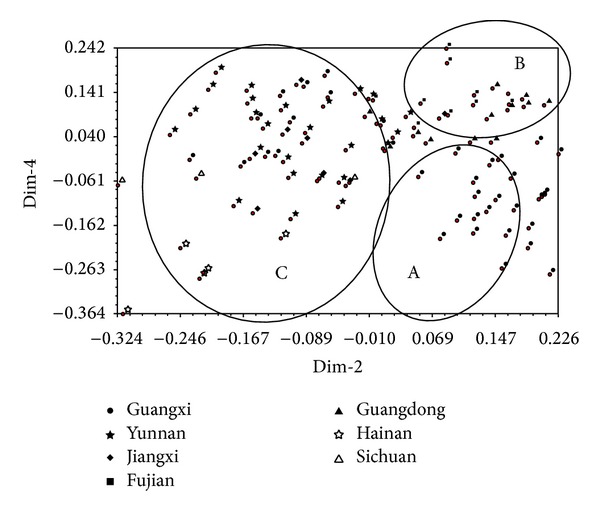
Principal components analysis of the 100* S. scitamineum* isolates (numbers 1–100) based on SP-SRAP data.

PCA analysis of the 100* S. scitamineum* isolates was performed based on SP-SRAP data by using the software NTSYS [30]. The results were shown in Figure 5. Amongst the 100 analysed isolates, 97 were assorted into three different groups, named as A, B, and C, except for the isolate number 24 from Guangxi, the isolate number 95 from Hainan, and the isolate number 98 from Sichuan. The group A includes 22 isolates from Guangxi Province. The group B includes 22 isolates, of which eight from Fujian, 11 from Guangdong, one from Guangxi, one from Yunnan, and one from Jiangxi. The group C includes 53 isolates, of which 12 from Guangxi, 25 from Yunnan, seven from Jiangxi, four from Hainan, two from Guangdong, two from Sichuan, and one from Fujian. It is notable that 20 out of 22 isolates in the group A were also included in the cluster I of UPGMA, 16 out of 22 in the group B were also included in the cluster II of UPGMA, and 47 out of 53 in the group C were also included in the cluster III of UPGMA (Figure 4). All the isolates in the group A were collected from Guangxi, the most of the isolates in the group B were collected from Fujian and Guangdong, and the isolates in the group C were composed of those from seven different provinces.

### 3.4. Genetic Diversity Analysis Based on the Combined ISSR and SP-SRAP Data Set

**Figure 6:**
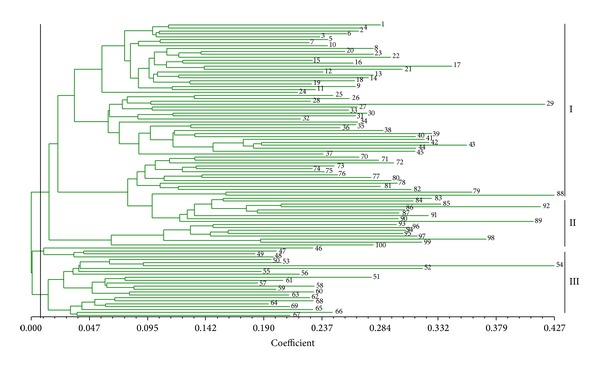
Dendrogram of the 100* S. scitamineum* isolates (numbers 1–100) generated by UPGMA based on the combined ISSR + SP-SRAP data set.

By combining the data of ISSR and SP-SRAP, a total of 274 bands were recorded, of which 257 (93.80%) were polymorphic on the 100* S. scitamineum* isolates. UPGMA cluster analysis based on these combined data revealed three clusters, named I, II, and III (Figure 6). Of the 100 isolates, 76 (76%) were grouped in cluster I, including all the isolates from Guangxi, Fujian, Guangdong, Hainan, and Sichuan, plus nine isolates from Yunnan and one isolate from Jiangxi. Only four isolates (4%) from Yunnan were grouped in cluster II. The remaining 20 isolates (20%), of which 13 were from Yunnan and seven from Jiangxi, were grouped into the cluster III. Comparable to those observed above, the isolates collected from the same region tend to be grouped into one cluster.

**Figure 7:**
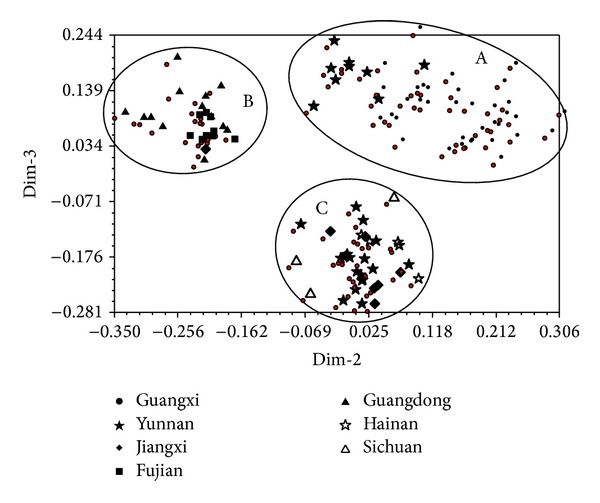
Principal components analysis (PCA) of* S. scitamineum* (numbers 1–100) based on the combined ISSR + SP-SRAP data set.

The results of PCA analysis of the 100* S. scitamineum* isolates based on the combined ISSR + SP-SRAP data set were shown in Figure 7. It showed that the group A contained 45 isolates, including all the 36 isolates sampled from Guangxi and nine isolates from Yunnan. The group B consisted of 23 isolates, including all the nine isolates sampled from Fujian, all the 13 isolates from Guangdong, and one isolate from Jiangxi. The group C contained 32 isolates, including 17 isolates from Yunnan, seven isolates from Jiangxi, all five isolates from Hainan, and all three isolates from Sichuan.

Compared to the results of UPGMA analysis, all 45 isolates in group A and all 23 isolates in group B were also grouped in the cluster I of UPGMA, while group C contained all four isolates in cluster II and all 20 isolates in cluster III, plus the remaining eight isolates in cluster I. The isolates isolated from the same district in a province tend to group together as revealed by both UPGMA and PCA analyses.

### 3.5. Population Genetic Analysis Based on the ISSR, SP-SRAP, and ISSR + SP-SRAP Data Set

Population genetic parameters for the 121 ISSR loci identified among the 100* S. scitamineum* isolates were estimated (Table 4). The results showed that the observed (*N*_*a*_) and effective (*N*_*e*_) numbers of alleles were higher in the population of Guangxi (*N*_*a*_ = 1.86, *N*_*e*_ = 1.48) and Yunnan (*N*_*a*_ = 1.85, *N*_*e*_ = 1.49) compared to other subpopulations. By comparison, the gene diversity index (*h*) and the Shannon's information index (*I*) were also relatively higher in the subpopulation of Yunnan (*h* = 0.29, *I* = 0.43) than those in the subpopulations of Hainan (*h* = 0.17, *I* = 0.25) and Sichuan (*h* = 0.17, *I* = 0.25), respectively. By using ISSR data set, the total gene diversity (*H*_*t*_) and gene diversities between subpopulations (*H*_*s*_) were estimated to be 0.34 and 0.22, respectively. Gene diversity and differentiation among populations (*G*_*st*_) were estimated to be 0.35, indicating that 35% of the total genetic variation was originated between populations, while gene flow (*Nm*) was estimated to be 0.94, indicating that lower rate of gene flow has occurred between populations.

Population genetic parameters for the 153 SP-SRAP loci identified among the 100 isolates was estimated (Table 4). Observed (*N*_*a*_) and effective (*N*_*e*_) numbers of alleles were higher in the subpopulations of Guangxi (*N*_*a*_ = 1.97, *N*_*e*_ = 1.57) and Yunnan (*N*_*a*_ = 1.96, *N*_*e*_ = 1.61) compared to other subpopulations. By comparison, the gene diversity (*h*) and the Shannon's information index (*I*) values were also relatively higher in the subpopulations of Yunnan (*h* = 0.35, *I* = 0.52) than those in the subpopulations of Hainan (*h* = 0.25, *I* = 0.37) and Sichuan (*h* = 0.26, *I* = 0.37), respectively. The total gene diversity (*H*_*t*_) and gene diversities between subpopulations (*H*_*s*_) values in SP-SRAP analysis were estimated to be 0.38 and 0.29, respectively. Gene diversity and differentiation among populations (*G*_*st*_) were estimated to be 0.22, indicating that 22% of the total genetic variation was originated between populations, while the gene flow (*Nm*) was estimated to be 1.78, indicating that higher rate of gene flow has occurred between populations.

Population genetic parameters for the 274 ISSR + SP-SRAP loci identified among the 100 isolates was estimated (Table 4). Observed (*N*_*a*_) and effective (*N*_*e*_) numbers of alleles were higher in the subpopulations of Guangxi (*N*_*a*_ = 1.92, *N*_*e*_ = 1.53) and Yunnan (*N*_*a*_ = 1.91, *N*_*e*_ = 1.56) compared to other subpopulations. The gene diversity (*h*) and the Shannon's information index (*I*) values were also relatively higher in the subpopulations of Yunnan (*h* = 0.32, *I* = 0.48), but relatively lower in the subpopulations of Hainan (*h* = 0.22, *I* = 0.32) and Sichuan (*h* = 0.22, *I* = 0.31), respectively. The total gene diversity (*H*_*t*_) and gene diversities between subpopulations (*H*_*s*_) values in ISSR + SP-SRAP analysis were estimated to be 0.26 and 0.36, respectively. The genetic diversity attributable to differentiation among populations (*G*_*st*_) was estimated to be 0.27, indicating that 27% of the total genetic variation was originated between populations, while the rate of gene flow (*N*_*m*_) was estimated to be 1.33, relatively lower than that of SP-SRAP (1.78) but higher than that of ISSR (0.94).

### 3.6. Analysis of ITS Regions for Six Pairs of Isolates with Low Genetic Similarity between Pairs

A total of 12 isolates in six pairs (number 45 and number 98, number 10 and number 89, number 18 and number 85, number 44 and number 47, number 55 and number 92, and number 63 and number 83), having low genetic similarity with the remaining pairs of the 100 isolates were selectedfor further PCR analysis of their rDNA-ITS, including ITS1, ITS2, and 5.8S rDNA, using the primer pair ITS1 and ITS4 [29]. PCR amplification revealed a single 755 bp fragment present in all the 12* S. scitamineum* isolates. The PCR products from each of these 12 isolates were sequenced. Sequence analysis showed that these isolates shared 94.09% to 96.98% similarity with an average of 96.34%, suggesting that rDNA-ITS regions contain lower sequence variations and may be not suitable for studying the genetic diversity of* S. scitamineum* in Mainland China.

## 4. Discussion and Conclusions

### 4.1. Confirmation of* S. scitamineum *Samples by ITS Sequence Analysis

Because of the limited genetic variability within the ITS region, ITS sequence data can normally define organisms at the genus level and resolve relationships between closely related species [21, 33]. In order to confirm the authenticity of each of the 100 tested* S. scitamineum* isolates, PCR amplifications of the ITS region were performed using the specific primer pair ITS1 and ITS4. As expected a single 755 bp fragment was obtained on each isolates. PCR amplifications with* S. scitamineum*—specific primer pair *b*E4 and *b*E8 yielded, as expected a single 420 bp fragment from all isolates.

These results showed that a single spore isolated from one smut whip can be used to generate a genetically fixed clone representing its original local population. Furthermore, sequence analysis of ITS regions of the six pairs of 12 isolates with lower genetic similarity indexes (from 0.4628 to 0.5627) showed very high sequence conservation between these isolates (sequence similarity ranged from 94.09% to 96.98%, with an average of 96.34%). This result confirms a previous observation by Singh et al. [6], that the sequence mutation rate in the ITS regions is very low among the smut pathogen strains.

### 4.2. Sugarcane Smut Race Classification by ISSR, SP-SRAP, and ISSR + SP-SRAP

Several races of* S. scitamineum* are known to exist but the race type is still poorly understood [4, 7, 34–37]. At the 24th International Society of Sugar Cane Technologists Congress held in Brisbane, the members of the Pathology Section agreed that only two races (1 and 2) of the sugarcane smut pathogen exist in the countries investigated, except in Chinese Taiwan, where a third race was identified. Molecular analysis has so far yielded data of little value to the race classification of sugarcane smut. Researchers were unable to separate into races based on polymorphisms detected by AFLP analysis [4, 33]. Using RAPD and SRAP analysis or a combination of the two, Que et al. were unable to separate 23* S. scitamineum* isolates into clusters based on smut race [7]. It demonstrated that the analysis of DNA from* S. scitamineum* from different geographic areas, by the RAPD, SRAP, and AFLP methods has so far yielded data of little value to the race classification of sugarcane smut.

Similar to the previous study [7], the results of this study showed no evidence that the smut could be divided into two or three races based on ISSR, SP-SRAP, and combined ISSR + SP-SRAP combined analysis. In the present study, smut isolate number 79 was classified as race 1 because it was isolated from sugarcane variety NCo310 which is susceptible to race 1 and resistant to race 2, respectively, based on the use of host differential for smut race identification. Isolates number 40 from YT86-368, number 80, and number 88 from YT89-113, and number 92 from YT96-86 can also be considered to be race 1 because they share a high genetic similarity to the isolate number 79. What should be stressed is that ROC16 is assumed to be smut resistant sugarcane variety in Guangxi Province. However, five smut isolates had been collected from ROC16 in Guangdong, Fujian and Jiangxi Provinces, indicating that its resistance to* S. scitamineum* requires further exploration.

### 4.3. Biogeographical Variation within the* S. scitamineum* Population Revealed by ISSR, SP-SRAP, and Combined ISSR + SP-SRAP Analysis

Ma et al. studied 102 isolates of* S. reilianum* collected from six provinces of China [38]. They found that the genetic diversity of* S. reilianum* was associated essentially with the geographic origins of the pathogen instead of their host genotypes [38]. The similar results were also obtained for* S. commune* [39]. In previous studies, the biogeographical variation within the population of* S. scitamineum* was studied by RAPD, SRAP, SSR, and AFLP methods [4, 7, 9]. Que et al. [7] found that the analyses of RAPD, SRAP, and combined RAPD-SRAP did not provide any evidence of the coevolution mechanism between* S. scitamineum* and its sugarcane host, while coevolutionary phenomena between pathogens and their hosts have been proposed in* Magnicellulatae* and* Podosphaera* [40, 41]. In the study of Que et al. [7], the analyses of RAPD, SRAP, and combined RAPD-SRAP on 23 isolates of smut pathogen suggested that the molecular evolution of* S. scitamineum* tends to be associated with their geographical origins, not to their host origins. The genetic diversity within a collection of 23 isolates of* S. scitamineum* from Mainland China was relatively high with a genetic dissimilarity index value ranging from 0.071 to 0.825 for RAPD analysis, from 0.045 to 0.947 for SRAP analysis, and from 0.026 to 0.838 for combined RAPD-SRAP analysis [7], while American and African* S. scitamineum* populations were extremely low in genetic diversity and all strains seem to belong to a single lineage according to a study of 142 single-teliospores using SSR markers [9]. In the present study, we used a larger number of strains (100) isolated from all the seven sugarcane cultivated provinces (20 districts) in Mainland China, of which 36 strains were isolated in Guangxi, the largest sugarcane-producing province in Mainland China. The average genetic similarity index estimated for the 100 isolates of* S. scitamineum* was 0.66 by ISSR and 0.62 by SP-SRAP. Our study showed that the genetic diversity among the Chinese smut pathogen population was relatively high and properly estimated by the two adopted methods with the SP-SRAP markers slightly more polymorphic than ISSR markers.

One of the aims of the present study was to estimate the genetic relationship between the subpopulations of* S. scitamineum* from different provinces of Mainland China and from different districts in Guangxi province which accounts for about 62% of the total sugarcane cultivated area in Mainland China. Also included in this study were 26 strains isolated from Yunnan province which is considered to be the plant genetic diversity center [42] because of the ecological diversity found in this province. The UPGMA cluster analyses based on the ISSR and SP-SRAP data sets revealed that the genetic diversity within the isolates from Guangxi, Fujian, and Guangdong was lower than those from Yunnan and Jiangxi (Figures 2 and 4). The isolates of Yunnan can be further divided into three to four subgroups by ISSR or by SP-SRAP analysis (Figures 2 and 4). Among all genetic diversity indexes adopted in this study, the highest values were observed for the isolates from Yunnan and the lowest values were observed for those from Hainan, indicating a higher level of genetic diversity in the subpopulation of Yunnan and a lower one in that of Hainan. This could be explained by the highly variable climatic and geographic factors of Yunnan province contrasting to those of Hainan. Our results in this study support the hypothesis that Yunnan province could be the biological genetic diversity center for* S. scitamineum*.

**Figure 8:**
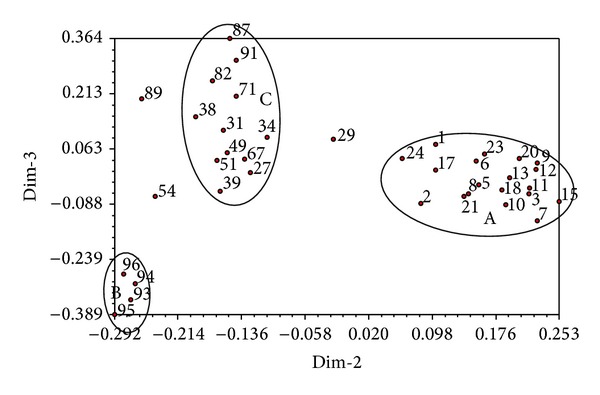
Principal components analysis of 38* S. scitamineum* isolates from ROC22 based on the combined ISSR + SP-SRAP data set. Notes: A: 19↑ Guangxi (1, 2, 3, 5, 6, 7, 8, 9, 10, 11, 12, 13, 15, 17, 18, 20, 21, 23, 24); B: 4↑ Hainan (93, 94, 95, 96); C: 15↑ Guangxi (27, 29, 31, 34), Yunnan (38, 39, 49, 51, 54), Jiangxi (67), Fujian (71), Guangdong (29, 82, 87, 91).

PCA analysis showed that the strains collected from the same region tend to be grouped together in the resulting plots (Figures 3, 5, and 7). In order to understand the genetic variability of the isolates isolated from one sugarcane genotype but collected from various geographical origins, we performed a PCA analysis on a total of 100 strains, including the 38 isolates from the sugarcane variety ROC22, 10 from Liucheng03-182, and seven from ROC16 using ISSR and SP-SRAP data sets. The results showed that the 38 isolates from ROC22 were divided into three different groups, named as A, B, and C (Figure 8). Among these 38 isolates, 19 isolates collected from Guangxi were classified into A, four isolates from Hainan into the group B, 15 isolates (four from Guangxi, five from Yunnan, four from Guangdong, one from Fujian, and one from Jiangxi) into the group C. Similar results were also obtained for the strains isolated from the sugarcane varieties Liucheng03-182 and ROC16 (data not shown).

Our results revealed the presence of geographical subdivisions among the 100* S. scitamineum* isolates, that is, the isolates of the smut pathogen collected from a same region tend to be clustered in the same group. Isolates from a same province were shown to share a high genetic similarity, while the isolates from different provinces were shown to be more genetically distant with lower genetic similarity values among them (Figures 2, 4, and 6; Table 4).

The highest genetic diversity was found within the subpopulation of Yunnan, in contrast to the lower diversity found among the isolates from Guangxi and Hainan. From the above results, we observed that the geographical origins contributed more to the clustering result than the host origins or sugarcane genotypes. This finding of geography-specific population of* S. scitamineum* is in agreement with the results of study on* S. commune* [39]. In recent years, the sugarcane varieties and seed-canes have been exchanged frequently without any quarantine and limitation between different provinces or regions in Mainland China and even introduced from Taiwan into Mainland China in large amount without quarantine for the past ten years, including the introduction of ROC22. The introduction of* S. scitamineum* together with the varieties or seed-canes may have increased the genetic variability of the pathogen into certain regions. Prevention and control of smut may be more difficult if the newly introduced strain may infect varieties resistant to endemic strains in the region.

### 4.4. Population Genetic Analysis Advances Our Understanding of the Genetic Structure of* S. scitamineum* Populations

Population genetic analysis, which can provide strong evidence about genetic diversity at the level of population or subpopulation, should be helpful for our understanding of the genetic structure of the studied populations [9, 31, 32, 43, 44]. Based on our ISSR, SP-SRAP, and ISSR + SP-SRAP data sets, the total gene diversity (*H*_*t*_) within the population of 100 isolates of smut pathogen was estimated to be 0.34, 0.22, and 0.26, respectively; the gene diversity between subpopulations (*H*_*s*_) was estimated to be 0.38, 0.29, and 0.36, respectively; the gene diversity attributable to differentiation among populations (*G*_*st*_) was estimated to be 0.35, 0.22, and 0.27, respectively; the gene flow (*Nm*) was estimated to be 0.94, 1.78, and 1.33, respectively. In each case, the estimated values of *H*_*t*_ and* Nm* are higher for SP-SRAP than for ISSR and ISSR + SP-SRAP, and the highest value of *H*_*s*_ was found for ISSR + SP-SRAP data set, while the highest value of *G*_*st*_ was found for ISSR data set. Therefore, the combined ISSR + SP-SRAP analysis should be more appropriate for the analysis of gene diversity between subpopulations (*H*_*s*_) and for the analysis of gene flow (*Nm*), SP-SRAP works better than ISSR and ISSR + SP-SRAP data set.

The gene flow parameter *Nm*, which means the product of the effective population number *N* and the rate of migration among populations, reflects the degree of genetic differentiation within populations. Methods for estimating how much gene flow occurs in natural populations can be divided into two categories; one is direct methods which involve estimating dispersal distances and reproductive success of individuals that disperse by direct observation [45]; the other one is indirect methods which rely on allele frequencies or differences in DNA sequences to estimate levels of gene flow that must have occurred in order to explain observed patterns [46]. In the present study, 100 strains can be assorted into seven subpopulations (Figures 2, 4, and 6), of which the distribution has a certain degree of discontinuity, which means that all these subpopulations should undertake natural genetic differentiation to some extent due to their various habitats. What should also be stressed is that the isolates in these subpopulations did not display any distinct phenotypic properties. Comparing the relative amounts of gene flow taking place among populations is the first step towards predicting the population structure of* S. scitamineum*. According to the value of proportion of gene diversity attributable to differentiation among populations (*G*_*st*_), the values of gene flow parameter *Nm* were estimated by indirect methods to be 0.94, 1.78, and 1.33, respectively, for ISSR, SP-SRAP, and combined ISSR + SP-SRAP analysis. All three analyses showed that there does exist significant gene flow between the seven* S. scitamineum* subpopulations in Mainland China. In addition to environmental factors that affect patterns of gene flow at the macrogeographic and regional scales, population structuring within ecological zones and habitats can potentially ruin efforts to drive genes into wild* S. scitamineum* populations [9, 32, 43, 44]. As there is always considerable variation within populations and among populations of sugarcane smut fungus, environmental heterogeneity may play an important role in these population variations [9, 47]. It can be inferred from above that the population genetic differentiation of sugarcane smut fungus depends at a large extent on the heterogeneity of habitats and is the result of its long-term adaptation to the ecological environment. Our conclusion is in agreement with the results of previous studies by Raboin et al. [9]and Zheng et al. [47].

## Figures and Tables

**Table 1:** Information about 100 strains of *S. scitamineum*.

No.	Source	Genotype of host
1	Futuo, Guangxi	ROC22
2	Futuo, Guangxi	ROC22
3	Futuo, Guangxi	ROC22
4	Futuo, Guangxi	YL17
5	Nanning, Guangxi	ROC22
6	Nanning, Guangxi	ROC22
7	Nanning, Guangxi	ROC22
8	Nanning, Guangxi	ROC22
9	Chongzuo, Guangxi	ROC22
10	Chongzuo, Guangxi	ROC22
11	Chongzuo, Guangxi	ROC22
12	Chongzuo, Guangxi	ROC22
13	Chongzuo, Guangxi	ROC22
14	Chongzuo, Guangxi	YC03-182
15	Chongzuo, Guangxi	ROC22
16	Tianyang, Guangxi	YC03-182
17	Tianyang, Guangxi	ROC22
18	Tiandong, Guangxi	ROC22
19	Tiandong, Guangxi	YT94-128
20	Tiandong, Guangxi	ROC22
21	Tiandong, Guangxi	ROC22
22	Tiandong, Guangxi	LC03-182
23	Nongkesuo, Guangxi	ROC22
24	Taocheng, Guangxi	ROC22
25	Hepu, Guangxi	YL6
26	Hepu, Guangxi	YT00-236
27	Yinhai, Guangxi	ROC22
28	Yinhai, Guangxi	GT02-901
29	Liucheng, Guangxi	ROC22
30	Liucheng, Guangxi	LC03-182
31	Liucheng, Guangxi	ROC22
32	Liucheng, Guangxi	LC03-1137
33	Laibing, Guangxi	LC03-182
34	Laibing, Guangxi	ROC22
35	Laibing, Guangxi	ROC16
36	Laibing, Guangxi	FN28
37	Baoshan, Yunnan	R6048
38	Baoshan, Yunnan	ROC22
39	Baoshan, Yunnan	ROC22
40	Baoshan, Yunnan	YT86-368
41	Baoshan, Yunnan	GT12
42	Nile, Yunnan	MT69-421
43	Nile, Yunnan	ROC16
44	Kaiyuan, Yunnan	Q170
45	Kaiyuan, Yunnan	GT11
46	Kaiyuan, Yunnan	LC03-182
47	Kaiyuan, Yunnan	MT69-421
48	Xinping, Yunnan	MT69-422
49	Xinping, Yunnan	ROC22
50	Lincang, Yunnan	LZ78-85
51	Lincang, Yunnan	ROC22
52	Lincang, Yunnan	LC03-182
53	Lincang, Yunnan	YZ03-103
54	Longchuan, Yunnan	ROC22
55	Longchuan, Yunnan	GT94-119
56	Luxi, Yunnan	LZ78-85
57	Luxi, Yunnan	YL6
58	Rili, Yunnan	LK80-279
59	Rili, Yunnan	YZ95-128
60	Yingjiang, Yunnan	LC03-182
61	Lianghe, Yunnan	LC03-182
62	Lianghe, Yunnan	LZ78-85
63	Nankang, Jiangxi	FN28
64	Nankang, Jiangxi	ROC16
65	Nankang, Jiangxi	YT91-600
66	Nankang, Jiangxi	LC03-182
67	Nankang, Jiangxi	ROC22
68	Nankang, Jiangxi	Co412
69	Nankang, Jiangxi	YZ95-128
70	Nankang, Jiangxi	GT02-351
71	Zhangzhou, Fujian	ROC22
72	Zhangzhou, Fujian	YT93-158
73	Zhangzhou, Fujian	YT93-158
74	Zhangzhou, Fujian	ROC16
75	Zhangzhou, Fujian	YT93-158
76	Zhangzhou, Fujian	ROC16
77	Fuzhou, Fujian	GT98-296
78	Fuzhou, Fujian	FN04-2861
79	Fuzhou, Fujian	NCo310
80	Zhanjiang, Guangdong	YT89-113
81	Zhanjiang, Guangdong	YT79-117
83	Zhanjiang, Guangdong	YT00-236
84	Zhanjiang, Guangdong	ROC16
85	Zhanjiang, Guangdong	ROC16
86	Zhanjiang, Guangdong	YT89-113
87	Zhanjiang, Guangdong	ROC22
88	Zhanjiang, Guangdong	YT89-113
89	Zhanjiang, Guangdong	ROC22
90	Zhanjiang, Guangdong	YZ03-194
91	Zhanjiang, Guangdong	ROC22
92	Zhanjiang, Guangdong	YT96-86
93	Haikou, Hainan	ROC22
94	Haikou, Hainan	ROC22
95	Haikou, Hainan	ROC22
96	Danzhou, Hainan	ROC22
98	Miyi, Sichuan	CZ6
99	Huili, Sichuan	CZ6
100	Dechang, Sichuan	CZ6

**Table 2:** List of the 7 selected ISSR primers and their PCR amplification results obtained on the 100 *S. scitamineum* isolates.

Primer	Sequence (5′-3′)		Bands	
		Total (No.)	Polymorphic (No.)	Polymorphic (%)
P5	(AG)_8_G	18	14	77.8
P6	(AG)_8_C	14	12	85.7
P10	(AG)_8_T	20	18	85.0
P21	(AG)_8_YA	20	19	95.0
P25	(GA)_8_YC	18	17	94.4
P26	(GA)_8_YT	19	17	89.4
P27	(GA)_8_YG	12	9	75.0

Total		121	105	
Mean			17.3	86.8

**Table 3:** List of the 7 selected SP-SRAP primers and their PCR amplification results obtained on the 100 *S. scitamineum* isolates.

Primer	Sequence (5′-3′)		Bands	
		Total (No.)	Polymorphic (No.)	Polymorphic (%)
P1	TGAGTCCAAACCGGATA	23	23	100.0
P2	TGAGTCCAAACCGGAAG	10	9	90.0
P3	TGAGTCCAAACCGGACA	20	20	100.0
P4	TGAGTCCAAACCGGACG	25	25	100.0
P5	TGAGTCCAAACCGGTAA	25	25	100.0
P6	GACTGCGTACGAATTAAT	23	23	100.0
P7	GACTGCGTACGAATTAAC	27	27	100.0

Total		153	152	
Mean			21	99.3

**Table 4:** Population genetic parameters estimated for different subpopulations of *S. scitamineum* collected from seven provinces by using the ISSR, SP-SRAP and ISSR + SP-SRAP data sets.

Geographic population^a^	Number of isolates	*N*_*a*_^b^	*N*_*e*_^c^	*h* ^d^	*I* ^e^
Guangxi	36	1.86^A^ 1.97^B^ 1.92^A+B^	1.48^A^ 1.57^B^ 1.53^A+B^	0.28^A^ 0.33^B^ 0.31^A+B^	0.42^A^ 0.50^B^ 0.47^A+B^
Yunnan	26	1.85^A^ 1.96^B^ 1.91^A+B^	1.49^A^ 1.61^B^ 1.56^A+B^	0.29^A^ 0.35^B^ 0.32^A+B^	0.43^A^ 0.52^B^ 0.48^A+B^
Guangdong	13	1.70^A^ 1.85^B^ 1.78^A+B^	1.36^A^ 1.50^B^ 1.44^A+B^	0.22^A^ 0.30^B^ 0.26^A+B^	0.34^A^ 0.45^B^ 0.40^A+B^
Fujian	9	1.54^A^ 1.75^B^ 1.65^A+B^	1.31^A^ 1.46^B^ 1.39^A+B^	0.19^A^ 0.27^B^ 0.23^A+B^	0.28^A^ 0.40^B^ 0.35^A+B^
Jiangxi	8	1.64^A^ 1.84^B^ 1.73^A+B^	1.38^A^ 1.52^B^ 1.45^A+B^	0.22^A^ 0.30^B^ 0.26^A+B^	0.34^A^ 0.46^B^ 0.40^A+B^
Hainan	5	1.43^A^ 1.63^B^ 1.54^A+B^	1.29^A^ 1.44^B^ 1.37^A+B^	0.17^A^ 0.25^B^ 0.22^A+B^	0.25^A^ 0.37^B^ 0.32^A+B^
Sichuan	3	1.39^A^ 1.58^B^ 1.49^A+B^	1.31^A^ 1.46^B^ 1.39^A+B^	0.17^A^ 0.26^B^ 0.22^A+B^	0.25^A^ 0.37^B^ 0.31^A+B^
Mean	14.3	1.63^A^ 1.79^B^ 1.72^A+B^	1.37^A^ 1.51^B^ 1.45^A+B^	0.22^A^ 0.29^B^ 0.26^A+B^	0.33^A^ 0.44^B^ 0.39^A+B^

^A^Genetic diversity of the 100 *S. scitamineum* isolates based on ISSR markers.

^B^Genetic diversity of the 100* S. scitamineum* isolates based on SP-SRAP markers.

^A+B^Genetic diversity of the 100* S. scitamineum* isolates based on ISSR and SP-SRAP markers.

^a^The 100 *S. scitamineum* isolates were devided into 7 subpopulations according to their geographic origins.

^b^Mean observed number of alleles.

^c^Mean effective number of alleles.

^d^Mean of Nei's gene diversity.

^e^Mean of Shannon's Information index.
